# Prehospital Care

**DOI:** 10.1155/2012/965480

**Published:** 2012-07-11

**Authors:** Stephen H. Thomas, Christopher Colwell, Jean-Claude Deslandes, Sophia Dyer, Jeffrey M. Goodloe

**Affiliations:** ^1^Department of Emergency Medicine, The University of Oklahoma, Norman, OK 13019-0390, USA; ^2^Department of Emergency Medicine, Denver Health and University of Colorado, Denver, CO 80217, USA; ^3^France's Urgence Pratique Formation, Ganges 34190, France; ^4^Department of Emergency Medicine, Boston University, Boston, MA 02215, USA

 The emergence of emergency medical services (EMS) as a realm within emergency medicine (EM) has recently been completed, with EMS gaining status as a boarded subspecialty. While there are excellent journals and a rich literature addressing EMS and out-of-hospital care, we embraced, with enthusiasm, the invitation of the editors to coordinate an EMS-related special issue on prehospital Care. The open access and online nature of the offers a unique chance for widespread attention to EMS, at a time when that attention is particular *a propos *given the new focus associated with subspecialty status.

 As guest editors, we bring to the issue's review process over a century of experience in ground EMS, air transport, and disaster operations. Our backgrounds include experience with urban and rural ground and air EMS systems all over the U.S. and abroad. Our responsibilities include involvement with ground and/or air EMS care in the USA east coast (S. Dyer, of Boston University), the USA west (C. Colwell, Denver Health and University of Colorado), and the U.S. southwest (J. Goodloe and Stephen Thomas of the University of Oklahoma). International experience comes from J. Deslandes (France's Urgence Pratique Formation).

The breadth of interests from our collective background has translated into a similarly broad range of topics covered in this special issue on prehospital care. The solicitation of manuscripts allowed for virtually any topic within prehospital and out-of-hospital care, and the manuscripts received were a testament to the excellence of work around the world, in our chosen fields. We believe that the papers that were ultimately approved for publication in this special issue represent important contributions to the state of the evidence in EMS as follows.

 A paper focuses on an EMS angle of a subject that has become one of the most important rooms in the house of medicine: quality. In his review of clinical performance indicators, M. J. El Sayed outlines EMS's place in the dialogue about quality measurement. His overview provides background discussion, education, and practical direction for EMS services.

 Another article takes on a clinical conundrum facing acute care providers every day: what's causing this patient's dyspnea? J. E. Gough and K. L. Brewer address the differentiation of various causes of dyspnea-differentiation that can be tricky even in the hospital setting. The divergence of therapeutic approaches to varying dyspnea etiologies means that the earlier the true causes can be identified, the more rapidly patients can receive appropriately tailored therapy. In their pilot study, J. E. Gough and K. L. Brewer suggest that peak expiratory flow rate may provide useful clues to allow earlier differentiation of two major causes of dyspnea.

 Air medical transport is one of the more controversial aspects of EMS. Unfortunately, both sides of the HEMS debate frequently mischaracterize the existing evidence, with the statement “there is very little data addressing HEMS outcomes.” In an attempt to provide an overview of the existing evidence, so that those with HEMS outcomes interest can get the original research and judge quality and impact for themselves, J. Hatfield et al. provide an annotated bibliography of HEMS outcomes research since 2007.

 Another paper addresses patient safety. C. T. Crowder et al. provide a look into a matter which has managed to elude attention despite the recent increased emphasis on patient safety. Rather than examine issues related to medical care, the study assesses the safety of patient transport. Specifically, F. J. Crowder et al. focus on stretcher-related problems and patient (and EMS crew member) safety issues related to stretcher misadventures.

 A different paper is a review of a potentially interesting possibility for improving drug and fluid delivery in the prehospital and disaster settings. Hyaluronidase, used a half-century ago for “hypodermoclysis,” fell out of favor decades ago due to immune reactions to the animal-based enzyme. With the relatively new availability of human recombinant hyaluronidase, the early literature suggests there might be a potential role for subcutaneous fluid administration in some prehospital settings. A. O. Arthur et al. outline the current state of the evidence and provide some directions for potential future research.

 In the paper of B. King et al., they continue the focus on disaster and mass casualty situations. In their report on a wind-caused collapse of a large tent at a city festival, the authors outline the benefits of planning and provide some important lessons learned for future planning.

 The review by L. J. Hamilton et al. changes focus to another area of importance in EMS: pediatric care. While most of the literature addressing pediatric prehospital care has understandably focused on general pediatrics and trauma situations, Hamilton et al. narrow the discussion to children with complex chronic conditions. While there are (thankfully) relatively few of these patient numbers, transports of children with chronic complex medical conditions can occupy a disproportionately large percentage of an EMS service runs. The experience and insights of L. J. Hamilton et al. should assist those EMS services and providers who care for this special patient population.

 A study by A. O. Arthur et al. changes focus to the other end of the age spectrum. With the aging of the population (especially in the US), any suggestive trend of a problem regarding older adults can be a harbinger of a major upcoming issue. Arthur et al. identify such a potential trend, potentially related to economic concerns, of a rise in the proportion of “transport refusal” patients who are geriatric. It is of course unknown whether economic or other identifiable factors are responsible for this finding, but the authors' preliminary findings should prompt further analysis of the issue.

 The special issue wraps up with an overview of HEMS research endpoints and potential benefits. The summary is intended not so much to make the case for (or against) HEMS as having outcomes benefits, as it is intended to highlight specific areas of investigation and focus for future efforts to define HEMS role in prehospital care.

 It has been our pleasure to bring this special issue to the readership of this journal. We hope that the literature in the edition helps to move the EMS world forward or at least inform debates and discussions regarding how to provide the best possible care in the out-of-hospital setting. Of course, we would be delighted to hear from you, the readers, as to comments or questions about any of the articles.

*Stephen H. Thomas* *Stephen H. Thomas* *Christopher Colwell* *Christopher Colwell* *Jean-Claude Deslandes* *Jean-Claude Deslandes* *Sophia Dyer* *Sophia Dyer* *Jeffrey M. Goodloe* *Jeffrey M. Goodloe*

